# Integrated Metabolomic and Transcriptomic Analyses to Understand the Effects of Hydrogen Water on the Roots of *Ficus hirta Vahl*

**DOI:** 10.3390/plants11050602

**Published:** 2022-02-24

**Authors:** Jiqing Zeng, Hui Yu

**Affiliations:** 1 Key Laboratory of South China Agricultural Plant Molecular Analysis, Genetic Improvement Guangdong Provincial Key Laboratory of Applied Botany, South China Botanical Garden, Chinese Academy of Sciences, Guangzhou 510650, China; 2 Key Laboratory of Plant Resource Conservation and Sustainable Utilization, Guangdong Provincial Key Laboratory of Applied Botany, South China Botanical Garden, Chinese Academy of Sciences, Guangzhou 510650, China; yuhui@scbg.ac.cn; 3 Southern Marine Science and Engineering Guangdong Laboratory (Guangzhou), Guangzhou 511458, China

**Keywords:** Wuzhimaotao (*Ficus hirta Vahl*), hydrogen, transcription factors, secondary metabolism, phytohormones signaling pathways, phenylpropanoid biosynthesis and metabolism, Chinese herbal medicine

## Abstract

Wuzhimaotao (*Ficus hirta Vahl*) is an important medicinal and edible plant in China. The extract from the roots of *Ficus hirta Vahl* contains phenylpropanoid compounds, such as coumarins and flavonoids, which are the main active components of this Chinese herbal medicine. In this study, we analyzed the transcriptomic and metabolomic data of the hydrogen-water-treated roots of *Ficus hirta Vahl* and a control group. The results showed that many genes and metabolites were regulated in the roots of *Ficus hirta Vahl* that were treated with hydrogen water. Compared with the control group, 173 genes were downregulated and 138 genes were upregulated in the hydrogen-rich water treatment group. Differential metabolite analysis through LC-MS showed that 168 and 109 metabolites had significant differences in positive and negative ion mode, respectively. In the upregulated metabolites, the main active components of Wuzhimaotao, such as the phenylpropane compounds naringin, bergaptol, hesperidin, and benzofuran, were found. Integrated transcriptomic and metabolomic data analysis showed that four and one of the most relevant pathways were over enriched in positive and negative ion mode, respectively. In the relationship between metabolites and DEGs, phenylpropanoid biosynthesis and metabolism play an important role. This indicates that phenylpropanoid biosynthesis and metabolism may be the main metabolic pathways regulated by hydrogen water. Our transcriptome analysis showed that most of the DEGs with |log2FC| ≥ 1 are transcription factor genes, and most of them are related to plant hormone signal transduction, stress resistance, and secondary metabolism, mainly phenylpropanoid biosynthesis and metabolism. This study provides important evidence and clues for revealing the botanical effect mechanism of hydrogen and a theoretical basis for the application of hydrogen agriculture in the cultivation of Chinese herbal medicine.

## 1. Introduction

Wuzhimaotao (*Ficus hirta Vahl*) is a common Chinese herbal medicine in the Lingnan area of China. It is a folk homologous plant of food and medicine, which is called Guangdong ginseng. It is used by Hakka people in southern China to treat diseases such as spleen deficiency, tuberculosis, weakness, rheumatism, night sweats, and agalactism [1,2,3]. Wuzhimaotao uses the roots of *Ficus hirta Vahl* as medicine. It is mainly distributed in Guangdong, Guangxi, Jiangxi, Fujian, Yunnan, Hong Kong of China, and Southeast Asian countries. The isolated and identified compounds in Wuzhimaotao mainly include coumarins, flavonoids, and volatile oil. Modern pharmacological studies show that these compounds have antioxidant, anti-inflammatory, antibacterial, antiviral, and antitumor effects [4].

In fact, the main pharmacodynamic components of Chinese herbal medicine are usually plant secondary metabolites. Studies shows that stress plays an important role in the formation of genuine medicinal materials [5]. When plants are infected by biological or abiotic factors, they can synthesize and accumulate a series of low relative molecular weight compounds with disease resistance through the expression of resistance genes in vivo to resist the invasion of pathogens. These substances are commonly known as phytoalexins. Phytoalexins are usually secondary metabolites, which are a large class of small molecular compounds produced by plants in the process of secondary metabolism [6]. Common secondary metabolites include alkaloids, flavonoids, terpenoids, anthraquinones, coumarins, and lignins. Stress conditions can promote the accumulation of plant secondary metabolites, and the accumulation of secondary metabolites can improve the stress resistance of plants. Therefore, the measures to improve plant stress resistance are expected to promote the accumulation of plant secondary metabolites in an effort to improve the genuine quality of traditional Chinese medicine [5]. The active components (coumarins, flavonoids, and volatile oil) of Wuzhimaotao are common plant secondary metabolites. The quality of the traditional Chinese medicine Wuzhimaotao and the content of its active components are expected to improve as stress conditions or other methods are used to improve its stress resistance. 

We found that hydrogen is involved in plant stress responses by regulating plant hormone signal transduction [7]. Hydrogen water treatment can improve the disease resistance of plants and the salt and drought resistance of rice seedlings. Stress treatment can also improve the ability of rice to release hydrogen [7]. Hydrogen water treatment can reduce the harm of heavy metal stress to plants [8,9,10]. We also found that hydrogen water can promote the growth of mung bean and rice roots and seedlings [7]. Therefore, we put forward the concept of hydrogen agriculture, i.e., using hydrogen water and other kinds of hydrogen fertilizer to promote the growth of crops and improve their stress resistance, in order to reduce the use of pesticides and chemical fertilizers, protect the ecological environment, and ensure food safety [11]. In addition, hydrogen fertilizers could be applied to the cultivation of traditional Chinese medicine to improve the content of effective components, yield, stress resistance, and, therefore, the quality of genuine medicinal materials.

In order to determine whether hydrogen molecules can increase the content of plant secondary metabolites and the molecular mechanism of hydrogen regulating the synthesis of secondary metabolites, the metabolome and transcriptome of Wuzhimaotao were analyzed.

## 2. Results

### 2.1. Metabolomic Changes Associated with Hydrogen-Water-Treated Roots of Ficus hirta Vahl

To investigate the metabolic pathways that were perturbed in the roots of *Ficus hirta Vahl* by the hydrogen water, we performed an integrated analysis of metabolomics and transcriptomics. Using liquid chromatography mass-spectrometry (LC-MS) for untargeted metabolomic profiles, a total of 12 samples was detected, including 6 samples of hydrogen-water-treated roots and 6 controls.

**Figure 1:**
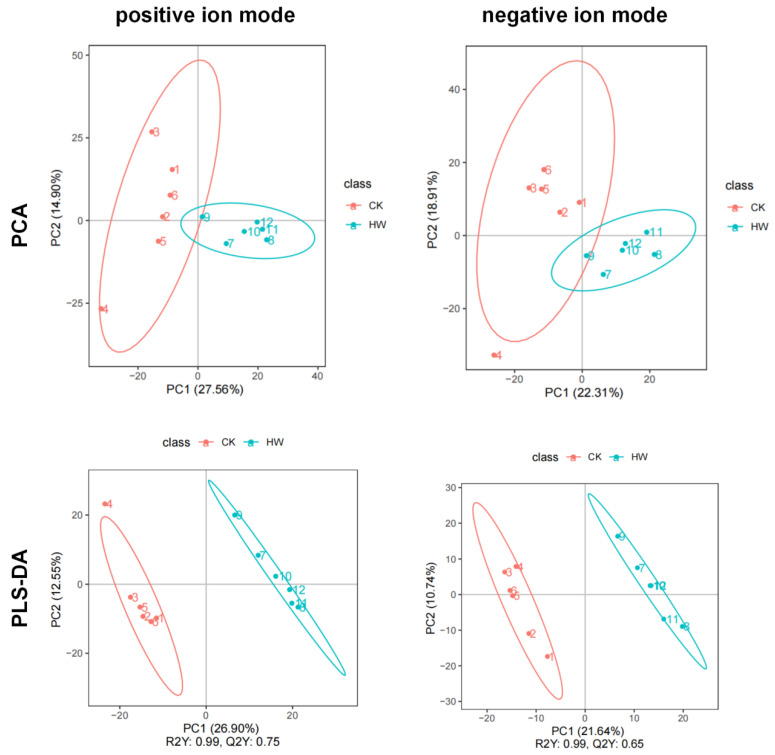
PCA and partial least squares discriminate analysis (PLS-DA) score chart of metabolite profiling data in positive ion mode and negative ion mode.

In order to further understand the differences between hydrogen-water-treated and control groups, we use Principal Component Analysis (PCA) for metabolite composition analysis, and we performed partial least squares discriminant analysis (PLS-DA) to sharpen the separation between groups of observations by rotating PCA components such that a maximum separation among classes was obtained and variables carrying the class separating information were located. Data of 1018 metabolites under the positive analysis ion mode and of 769 metabolites under the negative analysis ion mode were used in PLS-DA. Figure 1 shows that the score was 27.56% in ESI+ mode and 22.31% in ESI− mode. The hydrogen water treatment group could be clearly separated from the control group in the *x*-axis direction according to the PLS-DA model. The model quality was determined by parameters R2Y and Q2Y; R2Y = 0.99, Q2Y = 0.75 (Figure 1). The PLSDA score plot showed a clear separation between hydrogen-water-treated samples and control samples under the ESI− mode, in which R2Y and Q2Y values were greater than 0.5 (Figure 1). Therefore, the PLS-DA model with the first two components is sufficient to explain the difference between the hydrogen-water-treated samples and the control samples. 

**Figure 2:**
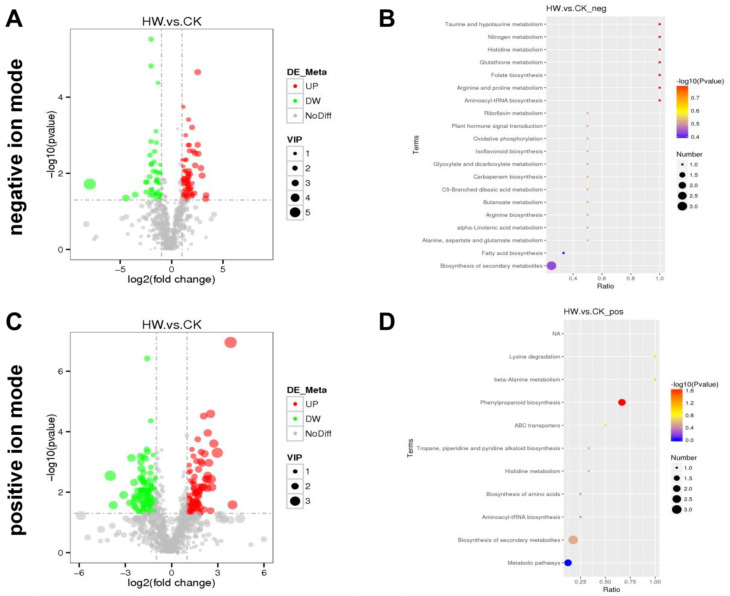
Metabolomics analysis of the hydrogen-water-treated group (HW) and the control group (CK). Volcano figure under negative ion mode (**A**) and positive ion mode (**C**); the green points represent the downregulated metabolites and the red points represent upregulating in the hydrogen water treated group. For the metabolite enrichment analysis under negative ion mode (**B**) and positive ion mode (**D**), the abscissa represents the ratio of the number of differential metabolites in the corresponding path to the total number of identified metabolites and the ordinate represents the impact of the path. The statistical significance is expressed in color gradient, and the size of the circle is directly proportional to the number of metabolites.

Significantly differentially expressed metabolites were identified according to the following criteria: PLS-DA VIP (variable importance in the projection) > 1, fold change > 2.0, and *p*-value < 0.05. A total of 1018 differentially expressed metabolites in hydrogen-water-treated samples compared to the control were identified in positive ion mode. A total of 168 significantly different metabolites were identified; 84 metabolites were upregulated while the other 84 metabolites were downregulated in hydrogen-water-treated samples. A total of 769 metabolites were identified in the negative ion mode. Compared with the control group, there were 109 significantly differentially expressed metabolites in the hydrogen water treatment group, including 64 upregulated metabolites and 45 downregulated metabolites (Table S1). Figure 2A,C show that all of these metabolites were included in the multivariate analysis. These results showed that hydrogen water treatment led to significant changes in root metabolism of *Ficus hirta Vahl*.

### 2.2. Pathway Enrichment Analysis 

In order to investigate the effect on related pathways regulated in roots of *Ficus hirta Vahl* by hydrogen water, pathway enrichment analysis was performed. Ten and twenty-three related metabolic pathways were identified in ES+ and ESI− mode, respectively. The results showed that the significantly different metabolites mainly associated with phenylpropanoid biosynthesis (*p* = 0.022) in ESI+ mode (Figure 2D). However, under the ESI− mode, lysine degradation (*p* = 0.159) and beta-alanine metabolism (*p* = 0.159) are close to significant (Figure 2B). The KEGG map of phenylpropanoid biosynthesis was shown in Figure S1.

### 2.3. Differentially Expressed Genes (DEGs) between Hydrogen Water Treatment Groups and Control Groups 

**Figure 3:**
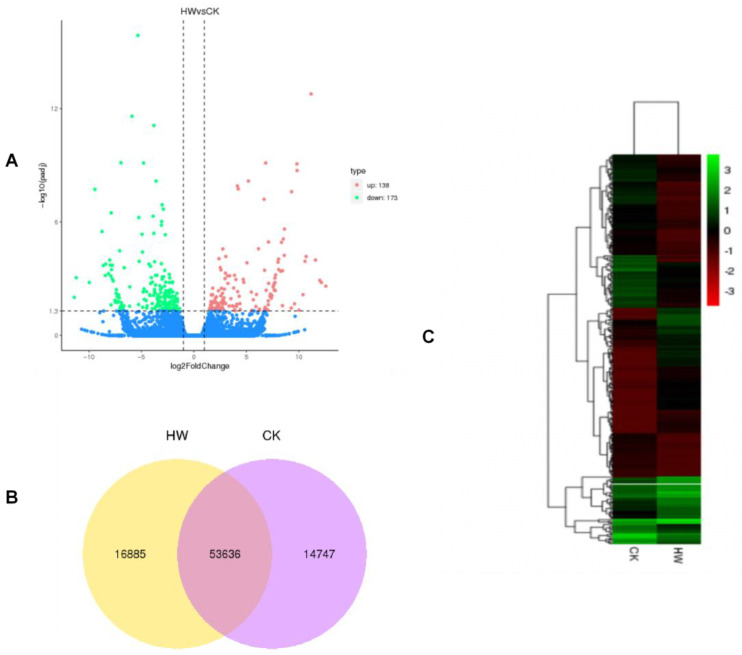
Cluster analysis of differentially expressed genes. (**A**) Volcano map of differentially expressed mRNAs. (**B**) Venn plot of the number of single genes between the two treatment groups. (**C**) Heat map of differentially expressed genes between hydrogen water treatment group and control group.

To understand the molecular basis of the metabolic pathway in roots of *Ficus hirta Vahl*, transcriptomes were analyzed to identify differentially expressed genes. RNA-seq data were from the hydrogen water treatment group and the control group, with three biological replicates respectively.We identified differentially expressed genes with the DESeq package by using the following criteria: fold change > 2 and adjusted *p*-value < 0.05. The obvious expressed unigenes with red and blue colors in two groups were clearly reflected in the Volcano plot. The results showed that 311 differentially expressed genes (DEG) were identified in roots of *Ficus hirta Vahl*, of which 138 were upregulated and 173 were downregulated (Figure 3A). Table S2 lists the upregulated and downregulated genes with significant differences between the two groups. The specificity and coexpression of mRNAs between the two groups are shown in the Venn diagram (Figure 3B). Transcripts clusters with significantly different expression patterns between the treatment group and the control group are highlighted on the heat map (Figure 3C). These results showed that hydrogen water treatment had a significant effect on transcription.

**Figure 4:**
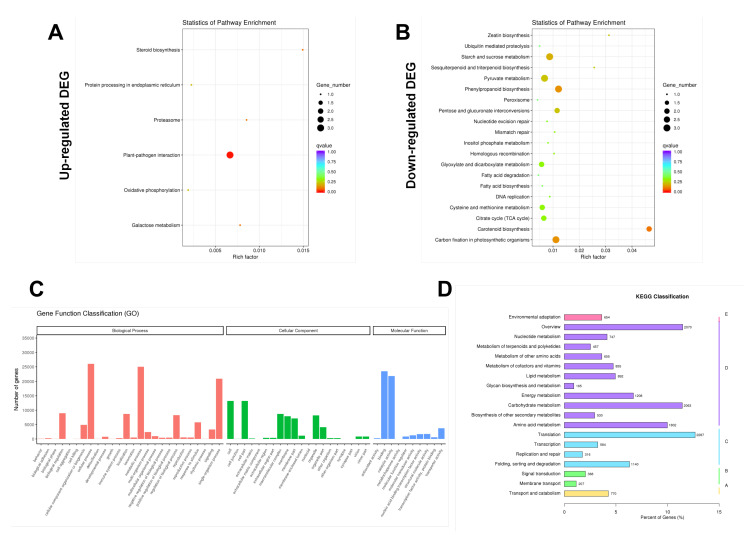
Functional analysis of DEGs between hydrogen water treatment group (HW) and control group (CK) based on Gene Ontology and KEGG pathway. (**A**) Scatter diagram of upregulated DEGs and enriched KEGG pathway; (**B**) scatter diagram of down regulated DEGs and enriched KEGG pathway; (**C**) enriched interaction network of GO terms in biological processes, cellular components, and molecular functions; (**D**) KEGG classification.

In order to further characterize DEGs, the KEGG pathway database was used for pathway analysis. The results showed that the transcripts regulated by hydrogen water in *Ficus hirta Vahl* roots could be mapped to signal pathways, such as carotenoid biosynthesis, phenylpropanoid biosynthesis, plant-pathogen interaction, carbon fixation in photosynthetic organisms, and starch and sucrose metabolism (Figure 4A,B and Figure S2). The gene ontology (GO) analysis of differentially expressed genes was classified based on GO annotation terms, and the gene expression pattern of the effect of hydrogen water on *Ficus hirta Vahl* roots was obtained. Figure 4C shows the enriched GO terms interaction network in biological processes, cellular components and molecular functions, and evaluates the relationship between differentially expressed genes.

### 2.4. Transcription Factors Related to Plant Hormone Signal Transduction, Stress Resistance, and Secondary Metabolite Biosynthesis in Roots of Ficus hirta Vahl 

**Figure 5:**
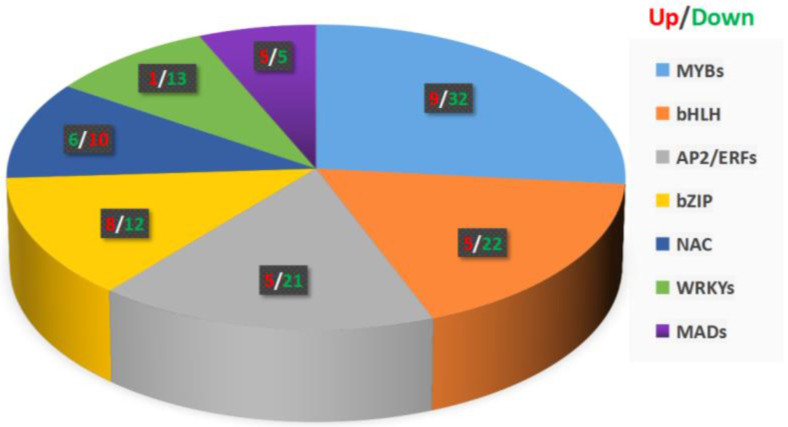
Transcription factors of phenylpropanoid biosynthesis in the roots of *Ficus hirta Vahl*.

Transcription factors participate in plant hormone signal transduction, stress resistance, and secondary metabolite biosynthesis by regulating the gene expression in plants. In our data, we found 625 transcription factors with different expression levels using iTAK software (http://itak.feilab.net/cgi-bin/itak/index.cgi/, accessed on 28 December 2021), of which, 168 transcription factors with significantly differentially expressed levels were related to phenylpropanoid biosynthesis and defense signaling (Table S3). Among these transcription factors, the most abundant were MYBs (41) and bHLHs (27), followed by AP2/ERFs (26), bZIPs (20), NACs (16), WRKYs (14), AUX/IAAs (12), MADs (10), and HSFs (2) (Figure 5). These transcription factors contribute to plant hormone signal transduction, stress resistance, and secondary metabolite biosynthesis in *Ficus hirta Vahl* roots. 

### 2.5. Integrated Analysis of the Mechanism of Hydrogen-Water-Treated Ficus hirta Vahl Root from Metabolomic and Transcriptomic Data 

**Figure 6:**
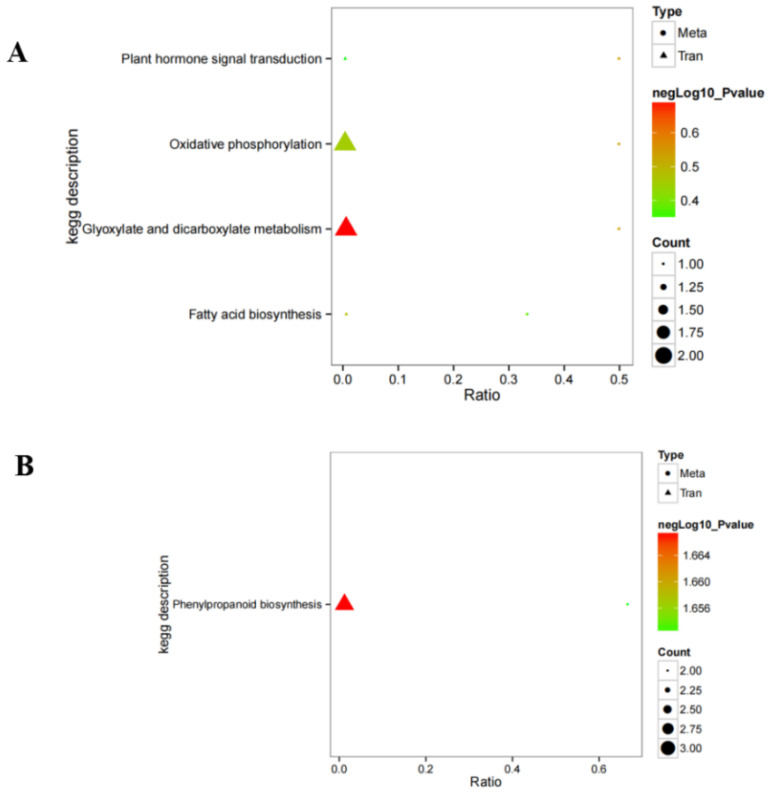
Integrated altered metabolic pathways in hydrogen-water-treated *Ficus hirta Vahl* roots. (**A**) negative ion mode; (**B**) positive ion mode.

**Figure 7:**
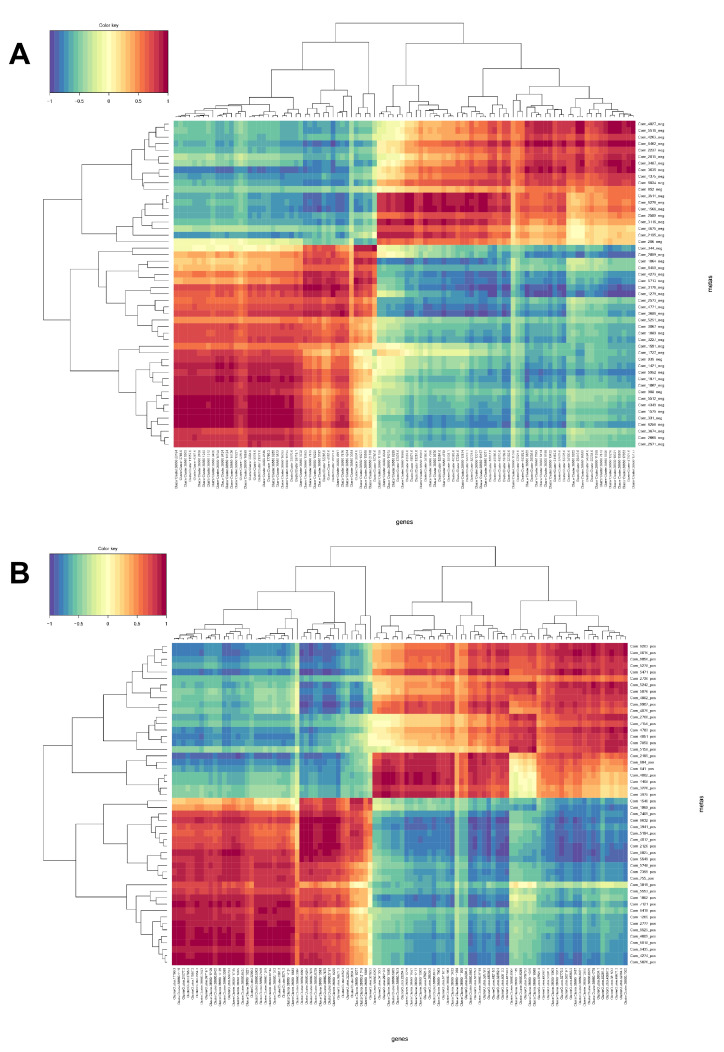
Integrative analysis based on metabolomics and transcriptomics data. (**A**) Negative ion mode. (**B**) Positive ion mode. The horizontal axis represents the differentially expressed gene ID number, and the vertical axis represents differential metabolite ID number. The blue depth indicates the intensity of the negative correlation. The red depth indicates the intensity of the positive correlation.

We further integrated metabolomic and transcriptomic data at the pathway level to explore the potential relationship between DEGs and metabolites. The results show that there are four enrichment pathways in ESI− mode, namely plant hormone signal transduction, oxidative phosphorylation, glyoxylic acid and dicarboxylic acid metabolism, and fatty acid biosynthesis (Figure 6), and only one enriched pathway under ESI+ mode, namely phenylpropanoid biosynthesis. Phenylpropanoid biosynthesis plays an important role in the potential relationship between DEGs and metabolites. These results suggest that phenylpropanoid biosynthesis might be the main metabolic pathway regulated by hydrogen water in *Ficus hirta Vahl* root. Figure 7 shows the heat map of comprehensive analysis between differential metabolite expression patterns and transcriptomics in ESI− mode and ESI+ mode. 

## 3. Discussion

Although the biological effects of hydrogen have long been noticed in botany and medicine [12,13,14], they attracted extensive attention only after 2007 [15]. Presently, it is understood that the mechanism of the hydrogen biological effect is that hydrogen has both antioxidant effects and signal molecule effects [16,17]. Our previous study found that hydrogen affects plant growth, development, and stress adaptation by participating in the regulation of plant hormone signaling pathways [7,18]. Because the improvement of plant stress resistance is often manifested in the accumulation of secondary metabolites [19,20,21], cultivation using hydrogen water may improve the accumulation of secondary metabolites, which may be conducive to improving the quality of traditional Chinese medicine. 

Wuzhimaotao is a famous traditional herbal medicine with homology of medicine and food in southern China that has been used for centuries. It is the dry root of *Ficus hirta*, a mulberry plant. The antioxidant, anti-inflammatory, antibacterial, antiviral, and antitumor effects of Wuzhimaotao have attracted more and more attention. It was reported that the main active components of Wuzhimaotao include coumarins, such as isopsoralen lactone, bergamot lactone, and psoralen, as well as flavonoids, such as apigenin and hesperidin [4]. It was reported that 70 compounds were isolated in Wuzhimaotao, of which 30 phenylpropanoid compounds were the most abundant [22]. The phenylpropane metabolic pathway in plants is an important pathway of secondary metabolism. All substances containing a phenylpropane skeleton are direct or indirect products of this pathway [23]. The phenylpropanoid biosynthetic pathway is activated under stress conditions, resulting in accumulation of various phenolic compounds which, among other roles, have the potential to scavenge harmful reactive oxygen species (ROS) [24,25,26]. In recent years, metabolomics integrated with transcriptomics has been widely used to investigate the biosynthesis of metabolites in plants [27,28]. In this study, hydrogen water was used to treat the famous Chinese southern medicine Wuzhimaotao, and then the metabolome and transcriptome were analyzed. Metabolomic analysis showed that there were a total of 277 significantly different metabolites between the hydrogen-water-treated group and the control group, including 148 upregulated and 129 downregulated metabolites. Among the upregulated metabolites, naringin (ID: Com_788_neg; fold change: 3.05), bergaptol (ID: Com_1406_neg; fold change: 2.28), hesperidin (ID: Com_3438_pos; fold change: 2.25), and a benzofuran (ID: Com_1053_neg; fold change: 2.19) were found (Table S1), all of which are phenylpropane compounds. As their congeners and derivatives, narigenin, bergapten, hesperidin, and benzofuran compounds of were isolated and identified as the active ingredients in Wuzhimaotao. Psoralen, a benzofuran derivative, is one of the most important active components of Wuzhimaotao. Recently, many benzofuran compounds were isolated from Wuzhimaotao, such as 3-[6-(5-O-β-D-glucopyranosyl) benzofuranyl] methyl propionate, (E)-3-[5-(6-hydroxy) benzofuranyl] propenoic acid, (E)-3-[5-(6-methoxy) benzofuranyl] propenoic acid, (Z)-3-[5-(6-O-β-D-glucopyranosyl) benzofuranyl] methyl propenoate, and S-3-[2,3-dihydro-6-hydroxy-2-(1-hydroxy-1-methylethyl)-5-benzofuranyl] methyl propionate [22]. Bergapten, psoralen, and other benzofurans belong to coumarins, and naringin and hesperidin belong to flavonoids. Coumarins and flavonoids are formed through the phenylpropanoid biosynthesis pathway. The results of the pathway enrichment analysis show that the significantly different metabolites were mainly associated with phenylpropanoid biosynthesis. The results show that hydrogen water treatment promoted the accumulation of active components in Wuzhimaotao, many of which were produced by phenylpropane synthesis. Therefore, planting Wuzhimaotao with hydrogen water may help to improve the content of the active components of Wuzhimaotao and improve the quality of traditional Chinese medicine. 

Transcriptome analysis showed that there were 311 significantly different DEGs between the hydrogen water treatment group and the control group, including 138 upregulated and 173 downregulated unigenes. KEGG pathway analysis found that transcripts regulated in the hydrogen-water-treated *Ficus hirta Vahl* roots could be mapped to signaling pathways, such as carotenoid biosynthesis, phenylpropanoid biosynthesis, and plant-pathogen interaction. The results of combined analysis of the metabolome and transcriptome showed that there were five enriched pathways: phenylpropanoid biosynthesis, plant hormone signal transduction, oxidative phosphorylation, glyoxylate and dicarboxylate metabolism, and fatty acid biosynthesis. The results show that hydrogen water treatment affected the phenylpropanoid biosynthesis and plant hormone signal transduction in *Ficus hirta Vahl* roots. Figure 4B shows that the DEGs of the phenylpropane synthesis pathway are downregulated, which seems to be inconsistent with the upregulation of coumarin and flavonoid metabolites shown by metabolome analysis. Comparing the KEGG enrichment obtained from metabolome and transcriptome analysis, we found that the phenylpropane synthesis pathway of the metabolome (Figure S1) is different from that of the transcriptome (Figure S2). Figure S2 shows that the metabolic pathways of coumarins and flavonoids are not downregulated. Therefore, there is no contradiction between the downregulation of the phenylpropane synthesis pathway shown by transcriptome analysis and the upregulation of coumarin and flavonoid metabolites shown by metabolome analysis. In addition, the integrated analysis of the metabolome and transcriptome showed that there was a significant positive or negative correlation between differential metabolites and differentially expressed genes (Figure 7). Therefore, the downregulation of genes related to the phenylpropane metabolic pathway is reflected in the downregulation of genes shown in Figure S2 and may also show that some negatively regulated transcription factor genes are downregulated.

The biosynthesis of secondary metabolites such as phenylpropanes is mainly regulated by transcription factors at the transcriptional level [29]. Recently, it has been found that six TF families are closely related to defense signaling. These six TF families are: AP2/ERF (APETALA2/ethylene responsive factor), bHLH (basic helix-loop-helix), MYB (myeloblastosis related), NAC (no apical meristem (NAM), Arabidopsis transcription activation factor (ATAF1/2), cup-shaped cotyledon (CUC2)), WRKY, and bZIP (basic leucine zipper) [30,31]. Interestingly, our transcriptome analysis showed that most of the DEGs with |log2FC| ≥ 1 are transcription factor genes. Among them, 41 MYB, 27 bHLH, 26 AP2/ERF, 20 bZIP, 16 NAC, 14 WRKY, 12 AUX/IAA, 10 MAD, and 12 HSF transcription factor genes were found. Clearly, these transcription factors are related to plant hormone signal transduction, stress resistance, and secondary metabolite synthesis. Since most of the transcription factors related to the phenylpropane metabolic pathway shown in Figure 6 are downregulated, we speculate that the downregulation of these transcription factors leads to the downregulation of the DEGs shown in Figure S2. It is also possible that some transcription factors play a negative regulation role and the down-regulation of transcription factors promote the coumarin and flavonoid metabolic pathways in the phenylpropane metabolic pathway. In addition, we did not rule out the possibility that the downregulation of other pathways of the phenylpropane metabolic pathway can promote the metabolism of coumarins and flavonoids.

Our previous study found that stress conditions can promote the release of hydrogen in plants [7]. Hydrogen can regulate the expression of transcription factor genes related to stress and plant hormone signal transduction, which suggests that hydrogen may be the secondary messenger under stress conditions. Since plant stress resistance and the accumulation of secondary metabolites are related to the regulation of plant hormones [7], these results suggest that hydrogen may act as a signal molecule to regulate the expression of transcription factor genes related to plant hormone and defense signal transduction, which is consistent with our previous research conclusions.

## 4. Materials and Methods

### 4.1. Plant Materials and Treatments

Twelve Wuzhimaotao (*Ficus hirta Vahl*) plants growing in South China Botanical Garden were selected and evenly divided into two groups, the treatment group and the control group. Hydrogen water was obtained following the method of Li et al. [32]. Purified hydrogen gas (99.99%, *v*/*v*) (Kedi Gas Chemical Co., Ltd., Foshan, China) was bubbled into 5 L of pure water at a rate of 200 mL min^−1^ for 3 h to obtain the saturated hydrogen water. The hydrogen concentration of hydrogen water was determined using a hydrogen portable meter (Trustlex Co., Ltd., ENH-1000, Yokohama, Japan). The treatment group was watered with hydrogen water (0.8 ppm) once a week for a total of 3 times, while the control group was watered with pure water. On the 15th day, the roots of *Ficus hirta Vahl* were collected, frozen with liquid nitrogen and stored at −80 °C before metabolite extraction and RNA-Seq.

### 4.2. Metabolite Extraction and Analysis

Each sample tissue (100 mg) was grounded with liquid nitrogen, and the homogenate was resuspended with precooled 80% methanol and 0.1% formic acid by well vortexing. The sample was placed on ice for 5 min and then centrifuged at 15,000 rpm at 4 °C for 5 min. Part of the supernatant was diluted with methanol to a final concentration of 60% with LC-MS grade water.

The samples were subsequently transferred to a fresh Eppendorf tube with a 0.22 μm filter and then were centrifuged at 15,000× *g* at 4 °C for 10 min. Finally, the filtrate was injected into the LC-MS/MS system analysis. Liquid sample (100 μL) and prechilled methanol (400 μL) were mixed by well vortexing.

The Vanquish UHPLC system (Thermo Fisher, MA, USA) and Orbitrap Q Exactive series mass spectrometer (Thermo Fisher, MA, USA) were used for LC-MS/MS analysis. The flow rate of sample injected into a Hyperil Gold column (100 × 2.1 mm, 1.9 μm) was 0.2 mL/min, and the linear gradient retention time was 16 min. Eluent A (0.1% FA in water) and eluent B (Methanol) were used for positive mode. Eluent A (5 mM ammonium acetate, pH 9.0) and eluent B (Methanol) were used for negative polarity mode. The setting of solvent gradient was: 2% B, 1.5 min; 2–100% B, 12.0 min; 100% B, 14.0 min; 100–2% B, 14.1 min; and 2% B, 16 min. Under the positive and negative polarity mode, the Q Exactive mass spectrometer was run with a spray voltage of 3.2 kV, the capillary temperature at 320 °C, a sheath gas velocity of 35arb, and an auxiliary gas velocity of 10arb.

For peak alignment, pickup, and quantification of each metabolite, Compound Discoverer 3.0 (CD3.0, Thermo Fisher) was used to process the raw data file generated by UHPLC-MS/MS. The main parameters were as follows: retention time tolerance was 0.2 min; the actual mass tolerance was 5 ppm; signal strength tolerance was 30%; the signal-to-noise ratio was 3 and minimum intensity was 100,000. Then, the peak intensity was normalized to the total spectral intensity. Normalized data can be used to predict molecular formulas based on molecular ion peaks, additive ions, and fragment ions. Then, in order to obtain accurate qualitative and relative quantitative results, the peaks were matched with the mzCloud (https://www.mzcloud.org/, accessed on 23 May 2020) and ChemSpider (http://www.chemspider.com/, accessed on 23 May 2020) databases. Statistical analyses were performed using the statistical software R (R version R-3.4.3), Python (Python 2.7.6 version), and CentOS (CentOS release 6.6). When the data did not conform to the normal distribution, the area normalization method was used for normal transformation. 

These metabolites were annotated using the HMDB database (http://www.hmdb.ca/, accessed on 23 May 2020), KEGG database (http://www.genome.jp/kegg/, accessed on 23 May 2020), and Lipidmaps database (http://www.lipidmaps.org/, accessed on 23 May 2020). Principal component analysis (PCA) and partial least squares discriminant analysis (PLS-DA) were performed in metaX. Statistical significance (*p*-value) was calculated by univariate analysis (*t*-test). The identification criteria of differential metabolites were VIP > 1, *p*-value < 0.05, and fold change ≥ 2 or FC ≤ 0.5. The metabolites of interest were filtered according to the log2 (FC) and-log10 (*p*-value) of metabolites to obtain the volcano map.

The data were normalized using the Z score of the intensity region of differential metabolites, and the clustering heat map was drawn using the phatmap software package in R language. Cor() in R language (method = pearson) was used to analyze the correlation between different metabolites. We used cor.mtest() in R language to calculate the statistically significant correlation between different metabolites. The *p*-value with statistical significance was <0.05, and the correlation diagram was drawn with the corrplot software package in R language. We used the KEGG database to study the function and metabolic pathways of these metabolites. The criteria for metabolic pathway enrichment of differential metabolites are as follows. The metabolic pathway enrichment of differential metabolites were performed, when ratio were satisfied by x/n > y/N, metabolic pathway were considered as enrichment, when *p*-value of metabolic pathway < 0.05, metabolic pathway were considered as statistically significant enrichment.

### 4.3. Total RNA Extraction, RNA Quantification and Qualification

Frozen samples were ground in liquid nitrogen. Total RNA was extracted using a TRIzol reagent. We used 1% agarose gel electrophoresis to monitor RNA degradation and pollution. The NanoPhotometer^®^ spectrophotometer (IMPLEN, Westlake Village, CA, USA) was used to check RNA purity. The RNA Nano 6000 Assay Kit of the Agilent Bioanalyzer 2100 system (Agilent Technologies, Santa Clara, CA, USA) was used to assess RNA integrity. 

### 4.4. Library Preparation for Transcriptome Sequencing

The total amount of RNA in each sample used as input material for RNA sample preparation was 1.5 µg. Following the manufacture’s recommendations and adding index codes to attribute sequences to each sample, a NEBNext^®^ Ultra™ RNA Library Prep Kit for Illumina^®^ (NEB, Ipswich, MA, USA) was used to generate sequencing libraries. In short, mRNA was purified from total RNA by poly-Toligo-attached magnetic beads. 

Under elevated temperatures, divalent cations were used for cleavage in the NEBNext First Strand Synthesis Reaction Buffer (5X). The first strand of cDNA was synthesized by random hexamer primer and M-MuLV Reverse Transcriptase (RNase H-). The second strand of cDNA was then synthesized using DNA Polymerase I and RNase H. The remaining overhangs were transformed into blunt ends by exonuclease/polymerase activities. After adenylation of 3’ ends of DNA fragments, NEBNext Adaptor with hairpin loop structure were ligated to prepare for hybridization.Library fragments were purified using the AMPure XP system (Beckman Coulter, Beverly, USA) to preferentially select cDNA fragments with a length of 250~300 bp. Then 3 µL of USER Enzyme (NEB, USA) was used with size-selected, adaptor-ligated cDNA at 37 °C for 15 min followed by 5 min at 95 °C before PCR. PCR was then performed with Phusion High-Fidelity DNA polymerase, Universal PCR primers, and Index (X) Primer. Finally, the PCR products were purified (AMPure XP system) on the Agilent Bioanalyzer 2100 system and the library quality was evaluated. 

### 4.5. Clustering and Sequencing (Novogene Experimental Department) 

A TruSeq PE Cluster Kit v3-cBot-HS (Illumia) was used to cluster the index-coded samples on a cBot Cluster Generation System according to the manufacturer’s instructions. The prepared libraries were then sequenced on the Illumina Hiseq platform, and paired end reads were generated.

### 4.6. Data Analysis

Quality control: Raw data (raw reads) in fastq format were processed through the internal Perl script. In this process, clean data (clean reads) were obtained by deleting the reads including adapter, poly-N, and low-quality reads from the raw data. In addition, Q20, Q30, GC content, and sequence repeat level of clean data needed to be calculated. All the downstream analyses were based on clean data with high quality. 

Transcriptome assembly: The left files (read1 files) in all libraries and samples were merged into a large left.fq file, and the right files (read2 files) were merged into a large right.fq file. Transcriptome assembly was based on the left.fq and right.fq, and was completed using Trinity [33]; min_kmer_cov was set to 2 by default, and all other parameters were also set by default. 

Gene function annotation: Gene functions were annotated based on Nr (NCBI nonredundant protein sequences), Nt (NCBI nonredundant nucleotide sequences), KOG/COG (Clusters of Orthologous Groups of proteins), Pfam (Protein family), Swiss-Prot, KO (KEGG Ortholog database), and GO (Gene Ontology) databases. 

Differential expression analysis: The differential expression of two groups of samples with biological duplication was analyzed by DESeq R package (1.10.1). DESeq provides statistical routines for determining differential expression in digital gene expression data using a model based on the negative binomial distribution. In order to control the error detection rate, the resulting *p* value was adjusted using the method of Benjamin and Hochberg. Genes found by DESeq were identified as differentially expressed genes if their adjusted *p* value was <0.05. The threshold of significant differential expression was set as q value < 0.005 and |log2 (foldchange)| > 1.

GO enrichment analysis: GO enrichment analysis of the differentially expressed genes (DEGs) was realized by the GOseq R packages based on Wallenius’ noncentral hypergeometric distribution [34].

KEGG pathway enrichment analysis: KEGG [35] is a database resource (http://www.genome.jp/kegg/, accessed on 28 December 2021) used to understand the advanced functions and utilities of biological systems, such as cells, organisms, and ecosystems, from molecular level information, especially large-scale molecular data sets generated by genome sequencing and other high-throughput experimental technologies. KOBAS [36] software was used to test the statistical enrichment of differentially expressed genes in the KEGG pathway.

## 5. Conclusions

In order to study the changes of overall metabolite spectrum related to the effects of hydrogen water on *Ficus hirta Vahl* roots, metabolomics and transcriptomics were analyzed by LC-ESI-MS/MS and RNA-seq techniques. Our study demonstrated that hydrogen water led to significant metabolic alterations in roots of *Ficus hirta Vahl*. The significantly upregulated metabolites included narigenin, bergapten, hesperidin, and benzofuran compounds. These active components of Wuzhimaotao are flavonoids and coumarins, which are produced through phenylpropane synthesis. By integrating transcriptomic data obtained by RNA-seq, we indicated that phenylpropanoid biosynthesis and metabolism may be the major metabolic pathways disturbed by hydrogen water. Additionally, we revealed that molecular hydrogen may regulate the expression of transcription factor genes related to plant hormone signal transduction, stress resistance, and secondary metabolite synthesis. This study provides important clues and evidence for understanding the botanical effect mechanism of hydrogen and a theoretical basis for the application of hydrogen agriculture in the cultivation of Chinese herbal medicine.

## Data Availability

Data is contained within the article or supplementary material.

## Supplementary Materials

The following are available online at https://www.mdpi.com/article/10.3390/plants11050602/s1, Figure S1: KEGG map of phenypropanoid biosynthesis pathway from metabolome analysis, Figure S2: KEGG map of phenypropanoid biosynthesis pathway from transcriptome analysis, Table S1: Metabolites differentially expressed in hydrogen rich water treated group as compared with control group, Table S2: DEG annotation, Table S3: Transcription factors related to plant hormone signal transduction, stress response, and secondary metabolite synthesis.

## References

[1] Song L.R., Hong X., Ding X.L., Zang Z.Y. (2001). Modern Chinese Pharmacy Dictionary.

[2] Pharmacopoeia Committee of the Ministry of Health of the People’s Republic of China (1997). Pharmacopoeia of the People’s Republic of China.

[3] Editorial Committee of Chinese Materia Medica, State Administration of Traditional Chinese Medicine (1999). Chinese Materia Medica.

[4] Lin H., Mei Q.X., Zeng C.Y. (2012). A survey of studies on chemical constituents and pharmacological activities of Wuzhimaotao (*Ficus hirta Vahl*). Pharm. Today.

[5] Huang L.Q., Guo L.P. (2007). Secondary metabolites accumulating and geoherbs formation under environmental stress. China J. Tradit. Chin. Mater. Med..

[6] Kuć J., Rush J.S. (1985). Phytoalexins. Arch. Biochem. Biophys..

[7] Zeng J., Zhang M., Sun X. (2013). Molecular hydrogen is involved in phytohormone signaling and stress responses in plants. PLoS ONE.

[8] Cui W., Fang P., Zhu K., Mao Y., Gao C., Xie Y., Wang J., Shen W. (2014). Hydrogen-rich water confers plant tolerance to mercury toxicity in alfalfa seedlings. Ecotoxicol. Environ. Saf..

[9] Cui W., Gao C., Fang P., Lin G., Shen W. (2013). Alleviation of cadmium toxicity in Medicago sativa by hydrogen-rich water. J. Hazard. Mater..

[10] Dai C., Cui W., Pan J., Xie Y., Wang J., Shen W. (2017). Proteomic analysis provides insights into the molecular bases of hydrogen gas-induced cadmium resistance in Medicago sativa. J. Proteom..

[11] Zeng J., Ye Z., Sun X. (2014). Progress in the study of biological effects of hydrogen on higher plants and its promising application in agriculture. Med. Gas Res..

[12] Renwick G.M., Giumarro C., Siegel S.M. (1964). Hydrogen metabolism in higher plants. Plant Physiol..

[13] Dole M., Wilson F.R., Fife W.P. (1975). Hyperbaric hydrogen therapy: A possible treatment for cancer. Science.

[14] Gharib B., Hanna S., Abdallahi O.M., Lepidi H., Gardette B., De Reggi M. (2001). Anti-inflammatory properties of molecular hydrogen: Investigation on parasite-induced liver inflammation. C. R. Acad. Sci. III.

[15] Ohsawa I., Ishikawa M., Takahashi K., Watanabe M., Nishimaki K., Yamagata K., Katsura K., Katayama Y., Asoh S., Ohta S. (2007). Hydrogen acts as a therapeutic antioxidant by selectively reducing cytotoxic oxygen radicals. Nat. Med..

[16] Li C., Gong T., Bian B., Liao W. (2018). Roles of hydrogen gas in plants: A review. Funct. Plant Biol..

[17] Russell G., Zulfiqar F., Hancock J.T. (2020). Hydrogenases and the Role of Molecular Hydrogen in Plants. Plants.

[18] Liu F., Li J., Liu Y. (2016). Molecular hydrogen can take part in phytohormone signal pathways in wild rice. Biol. Plant..

[19] Yang L., Wen K.S., Ruan X., Zhao Y.X., Wei F., Wang Q. (2018). Response of Plant Secondary Metabolites to Environmental Factors. Molecules.

[20] Ramakrishna A., Ravishankar G.A. (2011). Influence of abiotic stress signals on secondary metabolites in plants. Plant Signal. Behav..

[21] Ho T.T., Murthy H.N., Park S.Y. (2020). Methyl Jasmonate Induced Oxidative Stress and Accumulation of Secondary Metabolites in Plant Cell and Organ Cultures. Int. J. Mol. Sci..

[22] Chen J. (2017). The Research on the Bioactive Constituents of the Roots of Hairy Fig (*Ficus hirta Vahl*.). Master’s Thesis.

[23] Ouyang G., Xue Y. (1988). Physiological role and regulation of phenylpropanoid metabolism in plant. Plant Physiol. Commun..

[24] Sharma A., Shahzad B., Rehman A., Bhardwaj R., Landi M., Zheng B. (2019). Response of Phenylpropanoid Pathway and the Role of Polyphenols in Plants under Abiotic Stress. Molecules.

[25] Dixon R.A., Achnine L., Kota P., Liu C.J., Reddy M.S., Wang L. (2002). The phenylpropanoid pathway and plant defence—A genomics perspective. Mol. Plant Pathol..

[26] Korkina L. (2007). Phenylpropanoids as naturally occurring antioxidants: From plant defense to human health. Cell Mol. Biol..

[27] Lei Z., Zhou C., Ji X., Wei G., Huang Y., Yu W., Luo Y., Qiu Y. (2018). Transcriptome Analysis Reveals genes involved in flavonoid biosynthesis and accumulation in Dendrobium catenatum From Different Locations. Sci. Rep..

[28] Jiang T., Guo K., Liu L., Tian W., Xie X., Wen S., Wen C. (2020). Integrated transcriptomic and metabolomic data reveal the flavonoid biosynthesis metabolic pathway in *Perilla frutescens* (L.) leaves. Sci. Rep..

[29] Meraj T.A., Fu J., Raza M.A., Zhu C., Shen Q., Xu D., Wang Q. (2020). Transcriptional Factors Regulate Plant Stress Responses through Mediating Secondary Metabolism. Genes.

[30] Ng D.W., Abeysinghe J.K., Kamali M. (2018). Regulating the Regulators: The Control of Transcription Factors in Plant Defense Signaling. Int. J. Mol. Sci..

[31] Seo E., Choi D. (2015). Functional studies of transcription factors involved in plant defenses in the genomics era. Brief Funct. Genom..

[32] Li F., Hu Y., Shan Y., Liu J., Ding X., Duan X., Zeng J., Jiang Y. (2022). Hydrogen-rich water maintains the color quality of fresh-cut Chinese water chestnut. Postharvest Biol. Technol..

[33] Grabherr M.G., Haas B.J., Yassour M., Levin J.Z., Thompson D.A., Amit I., Adiconis X., Fan L., Raychowdhury R., Zeng Q. (2011). Full-length transcriptome assembly from RNA-Seq data without a reference genome. Nat. Biotechnol..

[34] Young M.D., Wakefield M.J., Smyth G.K., Oshlack A. (2010). Gene ontology analysis for RNA-seq: Accounting for selection bias. Genome Biol..

[35] Kanehisa M., Araki M., Goto S., Hattori M., Hirakawa M., Itoh M., Katayama T., Kawashima S., Okuda S., Tokimatsu T. (2008). KEGG for linking genomes to life and the environment. Nucleic Acids Res..

[36] Mao X., Cai T., Olyarchuk J.G., Wei L. (2005). Automated genome annotation and pathway identification using the KEGG Orthology (KO) as a controlled vocabulary. Bioinformatics.

