# Supplementary figures and images for: Integrated Metabolomic and Transcriptomic Analyses to Understand the Effects of Hydrogen Water on the Roots of Ficus hirta Vahl
Source: Plants (Basel). 2022 Feb 24;11(5):602. doi: 10.3390/plants11050602 (PMC8912395; doi:10.3390/plants11050602)

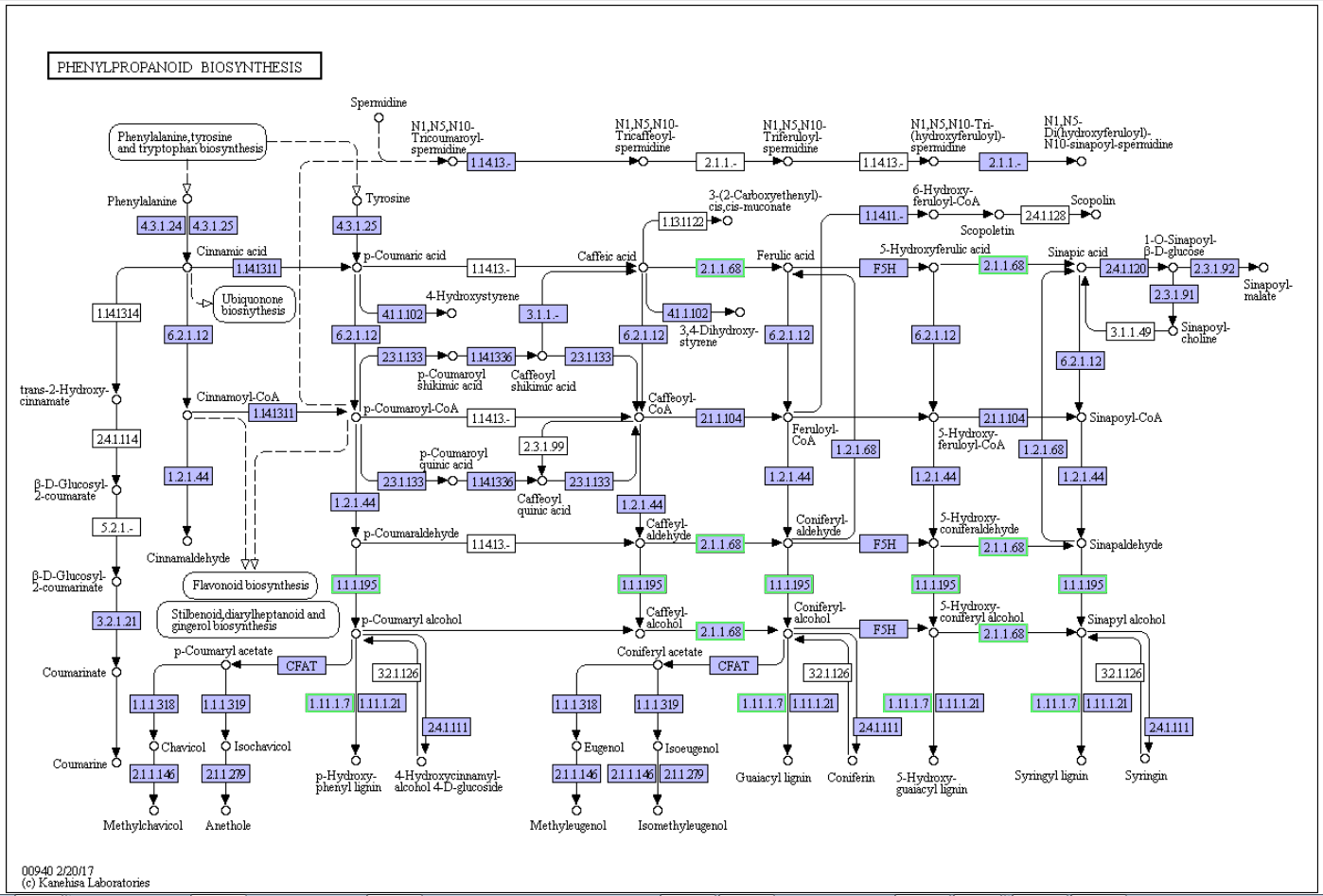

Supplement: Supplementary file 1 [file plants-11-00602-s001.zip › Figure S2.png]

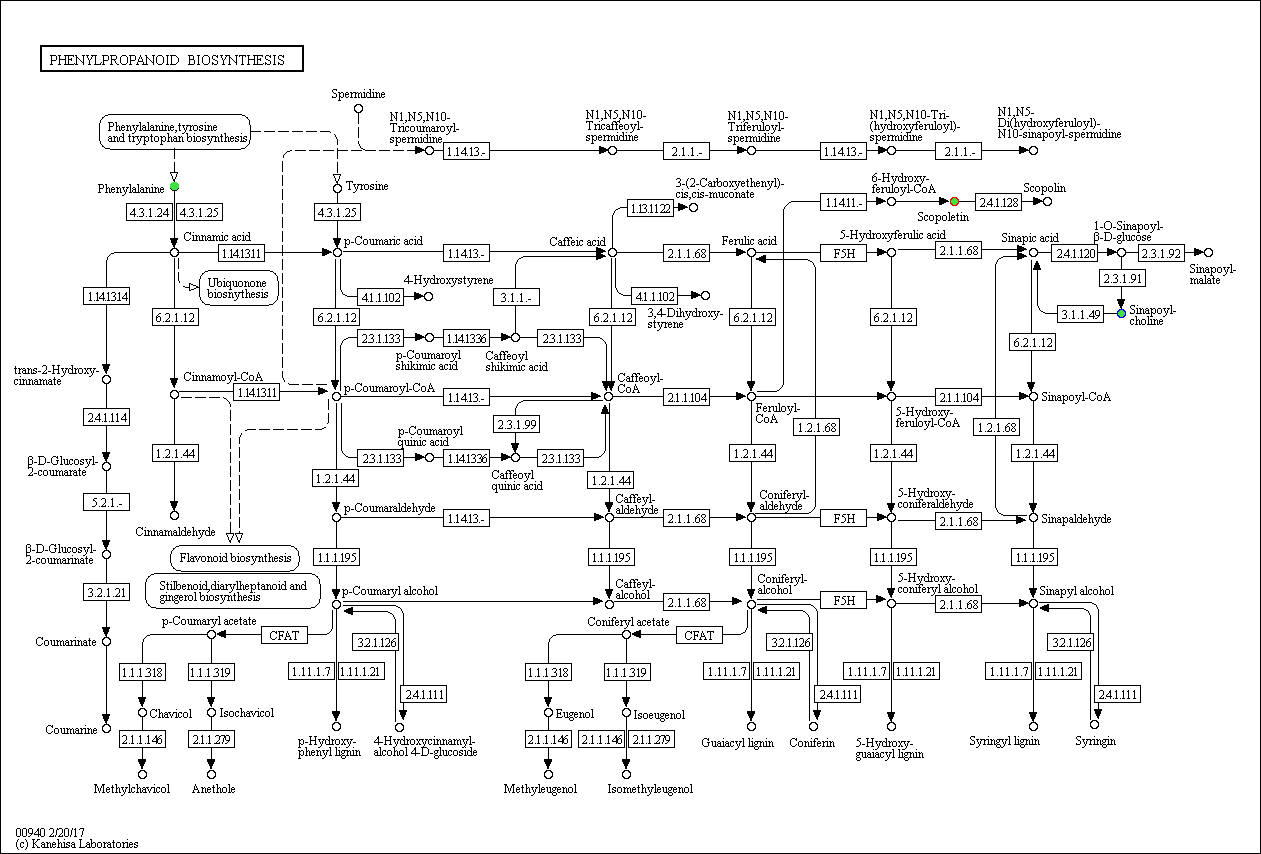

Supplement: Supplementary file 1 [file plants-11-00602-s001.zip › Figure S1.png]
